# Thyroid dysfunction and glycaemic control among Type 2 diabetes mellitus patients in Ghana: A comparative cross‐sectional study

**DOI:** 10.1002/edm2.447

**Published:** 2023-08-25

**Authors:** Samuel Asamoah Sakyi, Bright Ameyaw, Edwin Ferguson Laing, Richard Anthony, Richard K. Dadzie Ephraim, Alfred Effah, Afia Agyapomaa Kwayie, Ebenezer Senu, Enoch Odame Anto, Emmanuel Acheampong, Bright Oppong Afranie, Benjamin Amoani, Stephen Opoku

**Affiliations:** ^1^ Department of Molecular Medicine, School of Medicine and Dentistry Kwame Nkrumah University of Science and Technology Kumasi Ghana; ^2^ Laboratory Department Effia Nkwanta Regional Hospital Western Region Ghana; ^3^ Department of Internal Medicine Effia Nkwanta Regional Hospital Western Region Ghana; ^4^ Department of Medical Laboratory Technology University of Cape Coast Cape Coast Ghana; ^5^ Department of Medical Diagnostics, Faculty of Allied Health Sciences Kwame Nkrumah University of Science and Technology Kumasi Ghana; ^6^ School of Medical and Health Science Edith Cowan University Joondalup Australia

**Keywords:** endocrinopathies, glycaemic control, thyroid dysfunction, thyroid‐stimulating hormone, thyroxine, triiodothyronine, Type 2 diabetes

## Abstract

**Introduction:**

Thyroid disorders and diabetes mellitus coexist and are prevalent endocrinopathies among adult population. Thyroid dysfunction contributes to metabolic imbalances, increase beta‐cell apoptosis and glucose intolerance. There is paucity of data and contradicting findings on how thyroid dysfunction influence glycaemic control. Therefore, we evaluated thyroid dysfunction and glycaemic control among Type 2 diabetes mellitus (T2DM) patients in Ghana.

**Methods:**

A comparative cross‐sectional study was conducted among 192 T2DM patients from Effia Nkwanta Regional Hospital. Three consecutive monthly fasting plasma glucose (FBG) and glycated haemoglobin (HbA1c) were analysed and the results were classified as, moderate hyperglycaemia (MH) (FBG = 6.1–12.0 mmol/L, HbA1c < 7%), severe hyperglycaemia (SH) (FBG ≥ 12.1 mmol/L, HbA1c > 7%) and good glycaemic controls (GC) (FBG = 4.1–6.0 mmol/L, HbA1c < 7%). Thyroid‐stimulating hormone (TSH), free triiodothyronine (FT3) and free thyroxine (FT4), body mass index (BMI) and other clinical parameters were measured. Data analysis was done using R language version 4.0.2 and *p* < .05 was considered statistically significant.

**Results:**

There were no significant differences in age (years) between patients in the various glycaemic groups (*p* = .9053). The overall prevalence of thyroid disorders was 7.8% among T2DM patients. The prevalence of thyroid disorders was higher in patients with SH (11.7%) followed by those with MH (7.5%) and then those with GC (5.4%). Serum levels of TSH and FT3/FT4 ratio were significantly lower in T2DM patients with SH compared to those with MH and the GC (*p <* .0001). However, FT4 was significantly higher in SH patients compared to the good glycaemic controls (*p <* .01). The first tertiles of TSH [aOR = 10.51, 95% CI (4.04–17.36), *p* < .0001] and FT3 [aOR = 2.77, 95% CI (1.11–6.92), *p* = .0290] were significantly and independently associated with increased odds of hyperglycaemia.

**Conclusion:**

The prevalence of thyroid dysfunction is high in T2DM and increases with hyperglycaemia. Reduced TSH and T3 may worsen glycaemic control. Periodic monitoring of thyroid function should be incorporated into management guidelines among T2DM patients in Ghana.

## Graphical abstract

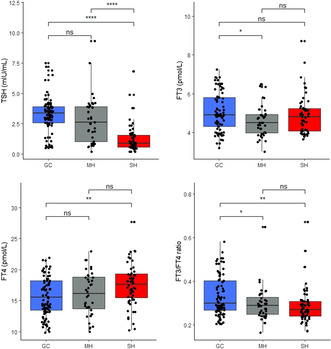

## 1 INTRODUCTION

Type 2 diabetes mellitus (T2DM) is a multifaceted metabolic disorder that results from insulin resistance and pancreatic beta‐cell dysfunction. T2DM is the most common form of diabetes mellitus (DM) and accounts for about 90% of all DM cases.^1^ It is a complex condition that arise from a combination of lifestyle and genetic factors.^2^, ^3^ About 415 million adults suffer from T2DM globally.^1^ In Ghana, the prevalence of T2DM is estimated to be 6% in the urban population.^4^ Comparable to diabetes, thyroid dysfunction emanates from uncontrolled hormone production and secretion. Hyperthyroidism and hypothyroidism which are the common thyroid disorders have been observed to be more common in T2DM patients compared to nondiabetic patients.^5^

Thyroid hormones regulate many cells and organs, and may affect protein, lipid and glucose metabolism, which in turn may exacerbate glycaemic control.^6^, ^7^ However, treatment guidelines for T2DM are not specific about how often thyroid function should be monitored in T2DM patients.^8^ Moreover, thyroid dysfunction can aggravate subclinical DM and results in hyperglycaemia increasing the risk of diabetic complications. Again, changes in thyroid hormone concentration can predispose prediabetes to overt T2DM.^8^

Consequently, thyroid disorders and diabetes mellitus coexist and are among the main endocrinopathies commonly found in the adult population.^9^ Uncontrolled blood glucose levels, frequency of hyperglycaemia and duration of diabetes has also been observed to affect the hypothalamic—anterior pituitary—thyroid axis.^10^ T2DM decreases thyroid‐stimulating hormone and impedes the production of thyroxine (T4) from triiodothyronine (T3).^11^ Thyroid hormones increase beta‐cell apoptosis and this could be a major element responsible for deterioration of glucose tolerance in thyrotoxicosis.^12^, ^13^ It is known that the discovery of abnormal thyroid hormones as well as other biochemical parameters in the initial stages of diabetes can assist in providing appropriate clinical intervention for patients.^2^, ^10^, ^14^ However, T2DM and thyroid dysfunction least investigated among Ghanaians. There is paucity of data and contradicting findings on how thyroid dysfunction influence glycaemic control among T2DM. This study therefore evaluated thyroid dysfunction and glycaemic control among T2DM patients in the Western Region of Ghana.

## 2 METHODOLOGY

### 2.1 Study design and setting

This cross‐sectional study was conducted at the Out Patient Department of the Effia Nkwanta Regional Hospital. Effia Nkwanta Regional Hospital (ENRH) is the major referral centre for residents of the Western region of Ghana. It is located in Sekondi‐Takoradi which is a twin city of with an estimated population of 560,000. Sekondi‐Takoradi is a cosmopolitan city with inhabitants coming from all over the country and accounts for ~24% of the region's total population.

### 2.2 Study population

The study population included 192 clinically diagnosed T2DM subjects, aged, 35 years and above, who attend diabetic clinic at the Effia Nkwanta Regional Hospital, Sekondi, Western Region. The diagnosis of T2DM was made based on American Diabetes Association (ADA) criteria [based on defective progressive insulin secretory on the background of insulin resistance using consistently high FPG results greater than or equal to 126 mg/dL (7.0 mmol/L) or random plasma glucose (RPG) results greater than or equal to 200 mg/dL (11.1 mmol/L)] and confirmed by HbA1c.^15^ Patients were confirmed as T2DM patients if they consistently meet the criteria over a period of 3 months. Patients with known Type 1 diabetes (diagnosed based on high plasma glucose in addition to autoimmune indicators) such as autoantibodies to insulin, autoantibodies to glutamate decarboxylase (GAD65) were excluded. Again, pregnant women, seriously ill diabetic patients, those with history of thyroid diseases, patients on thyroid medication and those who were unwilling to give consent were excluded from the study. Participants were categorized into three groups; patients with good glycaemic control (GC), moderate hyperglycaemia (MH) and those with severe hyperglycaemia (SH). GC were those participants whose HbA1c was <7% and FBG levels were within the normal range (4.1–6.0 mmol/L),^16^ during the past 3 months. MH were patients whose HbA1c was <7% and FBG results over the previous 3 months are between 6.1 and 12.0 mmol/L. SH were participants whose HbA1c was >7% and average FBG results over the previous 3 months are ≥12.1 mmol/L according to their records and clinical history from their folders.

### 2.3 Ethical consideration

Ethical approval (CHRPE/AP/064/16) for the study was obtained from the Committee on Human Research Publication and Ethics of the School of Medical Sciences, Kwame Nkrumah University of Science and Technology/Komfo Anokye Teaching Hospital and ENRH before the research was conducted. Written informed consent was obtained from each participant before administration of the standard structured questionnaire and collection of blood samples. Qualified participants were made to fill and sign or thumb print a consent form before recruiting them into the study. Confidentiality of participants was assured and all data were handled anonymously.

### 2.4 Anthropometric measurements

Anthropometric measurements for height and weight were taken from each participant and were used for the calculation of the body mass index (BMI) by dividing weight (kg) by height squared (m^2^). Their BMI was stratified into four groups: under‐weight (BMI < 18.5 Kg/m^2^), normal (BMI between 18.5 and 24.9 Kg/m^2^), overweight (BMI between 25 and 29.9 Kg/m^2^) and obese (BMI ≥ 30 Kg/m^2^).

### 2.5 Blood pressure measurement

Blood pressure (BP) was measured from each subjects using sphygmomanometer. This was measured three times, and the average reading was recorded. Individuals were deemed hypertensive if they were taking antihypertensive medications, or self‐reported a diagnosis of hypertension, or systolic pressure reading was above 140 mm Hg, or diastolic pressure reading was above 90 mm Hg, or combinations of these features.^17^

### 2.6 Blood sample collection and laboratory analysis

Six millilitres (6 mL) of venous blood sample were drawn from each participant after 8–12 h overnight fast and were dispensed into EDTA, fluoride and serum separator tubes, respectively. The fluoride and serum separator gel tubes were centrifuged at 3000 rpm for 5 min to separate cells from plasma and serum, respectively. The EDTA‐anticoagulated blood was used for HbA1c measurement whereas fluoride plasma was used to measure fasting blood glucose. Serum obtained from the separator gel tube was pipetted into cryotubes and stored at −70°C and used for thyroid function tests (TFT).

### 2.7 Classification of thyroid disorders

Thyroid dysfunction was considered if a participant's thyroid hormone level fell outside the reference range (TSH normal range: 0.4–5.5 μIU/mL, FT3 normal range: 2.8–7.3 pmol/L, FT4 normal range: 8.5–22.5 pmol/L). Participants with high TSH, and low FT3 and FT4 were classified as primary hypothyroidism and those with had elevated TSH, but with normal FT3 and FT4 were classified as subclinical hypothyroidism.^18^, ^19^, ^20^ Similarly, participants who had low TSH, and high FT3 and FT4 were classified as having primary hyperthyroidism and those who had low TSH but with normal FT3, and FT4 were classified as having subclinical hyperthyroidism.^21^, ^22^ Moreover, participants who had low or normal TSH, but had low FT3 and FT4 were classified as secondary hypothyroidism.^23^, ^24^

### 2.8 Statistical analyses

Statistical analysis was performed using R Language version 4.0.2.^25^ Data were screened for normality using the Kolmogorov–Smirnov test. Data were reported as means with standard deviations for continuous variables and as frequencies (percentages) for categorical variables. Association between categorical variables was performed using the Pearson's chi‐squared test. The one‐way ANOVA followed by Tukey's post hoc multiple comparison test was performed to compare differences in thyroid hormones between the good glycaemic controls (GC), moderate hyperglycaemia (MH) and severe hyperglycaemia (SH). The binary logistic regression analysis was used to determine the association between thyroid hormones and hyperglycaemia. Statistical significance was accepted at *p* < .05 for all comparisons.

## 3 RESULTS

### 3.1 Sociodemographic characteristics of the study participants

The statistical analysis included a total of 192 participants comprising of 100 T2DM patients (60 with SH and 40 with MH) and 92 GC. There were no significant differences in age between the good glycaemic controls (52.8 ± 7.8 years), moderate hyperglycaemia (53.4 ± 7.4 years) and severe hyperglycaemia groups (52.9 ± 7.7 years; *p* = .9053). Although females were more than their male counterparts in each of the glycaemic groups, there was no statistically significant difference (*p* = .068). Again, marital status, residence and exercise activity were not significantly different across the three groups (*p* > .05). We however observed that the ethnicity (*p =* .0288), education level (*p* < .0001), type of medication (*p* = .0005), duration of diabetes (*p* = .0080) and duration of treatment (*p* = .0080) were significantly associated with hyperglycaemia. Again, there was statistically significant difference in BMI between the three groups. It was however noted that the SBP (*p* = .7880) and DBP (*p* = .3637) did not differ significantly across the three groups. Table 1 displays the sociodemographic characteristics of the study participants.

**TABLE 1:** Sociodemographic characteristics of the study participants.

Parameters	Good glycaemic control (*n* = 92)	Moderate hyperglycaemia (*n* = 40)	Severe hyperglycaemia (*n* = 60)	*p*‐Value
*Age(years) (mean ± SD)*	52.8 ± 7.8	53.4 ± 7.4	52.9 ± 7.7	.9053
*Gender*				.0680
Male	34 (36.9)	7 (17.5)	22 (36.7)	
Female	58 (63.1)	33 (82.5)	38 (63.3)	
*Residence*				.2776
Rural	20 (21.7)	10 (25.0)	20 (33.3)	
Urban	72 (78.3)	30 (75.0)	40 (66.7)	
*Marital status*				.1590
Married	61 (66.3)	20 (50.0)	40 (66.7)	
Unmarried	31 (33.7)	20 (50.0)	20 (33.3)	
*Ethnicity*				**.0288**
Akan	73 (79.3)	39 (97.5)	49 (81.7)	
Others	19 (20.7)	1 (2.5)	11 (18.3)	
*Exercise*				.2123
Yes	62 (67.4)	30 (75.0)	35 (58.3)	
No	30 (32.6)	10 (25.0)	25 (41.7)	
*Level of education*				**<.0001**
No education	17 (18.5)	2 (5.0)	16 (26.7)	
Basic	31 (33.7)	32 (80.0)	40 (66.7)	
Secondary	34 (36.9)	3 (7.5)	3 (5.0)	
Tertiary	10 (10.8)	3 (7.5)	1 (1.7)	
*Type of medication*				**.0005**
OHA	50 (54.3)	16 (40.0)	44 (73.3)	
Insulin	22 (23.9)	13 (32.5)	1 (1.7)	
OHA + insulin	20 (21.7)	11 (27.5)	15 (25.0)	
*Duration of DM (years)*				**.0080**
0–5	52 (56.5)	14 (35.0)	21 (35.0)	
6–10	31 (33.7)	17 (42.5)	21 (35.0)	
>10	9 (9.8)	9 (22.5)	18 (30.)	
*Treatment duration (years)*				**.0080**
0–5	52 (56.5)	14 (35.0)	21 (35.0)	
6–10	31 (33.7)	17 (42.5)	21 (35.0)	
>10	9 (9.8)	9 (22.5)	18 (30.)	
SBP (mmHg)	131.3 ± 20.7	133.3 ± 18.5	133.3 ± 20.5	.7880
DBP (mmHg)	81.9 ± 9.4	83.3 ± 10.6	84.3 ± 11.8	.3637
BMI (kg/m^2^)	28.02 ± 4.1	30.3 ± 5.5	28.8 ± 4.7	**.0401**

*Note*: *p*‐Values computed by the chi‐squared test and the one‐way ANOVA.

Bold values indicate statistically significant *p*‐value (*p* < .05).

Abbreviations: BMI, body mass index; DBP, diastolic blood pressure; DM, diabetes mellitus; OHA, oral hypoglycaemic agent; SBP, systolic blood pressure.

### 3.2 Prevalence of thyroid disorders among T2DM patients

The overall prevalence of thyroid disorders was 7.8% among the T2DM patients. The prevalence of thyroid dysfunction was higher in SH (11.7%) followed by MH (7.5%) and the GC (5.4%). Of the total study participants 8 (4.2%) exhibited subclinical hypothyroidism. Five representing 5.4% of the 92 glycaemic controls had subclinical hypothyroidism. Two representing 5.0% of the 40 participants with moderate hyperglycaemia were sub‐clinically hypothyroid and 1 (1.7%) of the 60 severe hyperglycaemic participants were also sub‐clinically hypothyroid. In the good glycaemic control group, there were 5 (5.4%) participants with subclinical hypothyroidism, 2 (5.0%) moderate hyperglycaemia and 1 (1.7%) severe hyperglycaemia had subclinical hypothyroidism (Table 2).

**TABLE 2:** Prevalence of thyroid disorders among study participants.

Thyroid disorder	Total (192)	Good glycaemic control (*n* = 92)	Moderate hyperglycaemia (*n* = 40)	Severe hyperglycaemia (*n* = 60)
Subclinical hypothyroidism	8 (4.2)	5 (5.4)	2 (5.0)	1 (1.7)
Subclinical hyperthyroidism	6 (3.1)	0 (0.0)	1 (2.5)	5 (8.3)
Primary hyperthyroidism	1 (0.5)	0 (0.0)	0 (0.0)	1 (1.7)
**Total thyroid disorder**	**15 (7.8)**	**5 (5.4%)**	**3 (7.5%)**	**7 (11.7%)**
Normal thyroid	177 (92.2)	87 (94.6)	37 (92.5)	53 (88.3)

*Note*: Data presented as frequency and percentages (%).

Bold values indicate statistically significant *p*‐value (*p* < .05).

### 3.3 Comparison of thyroid hormones between the study groups

**FIGURE 1:**
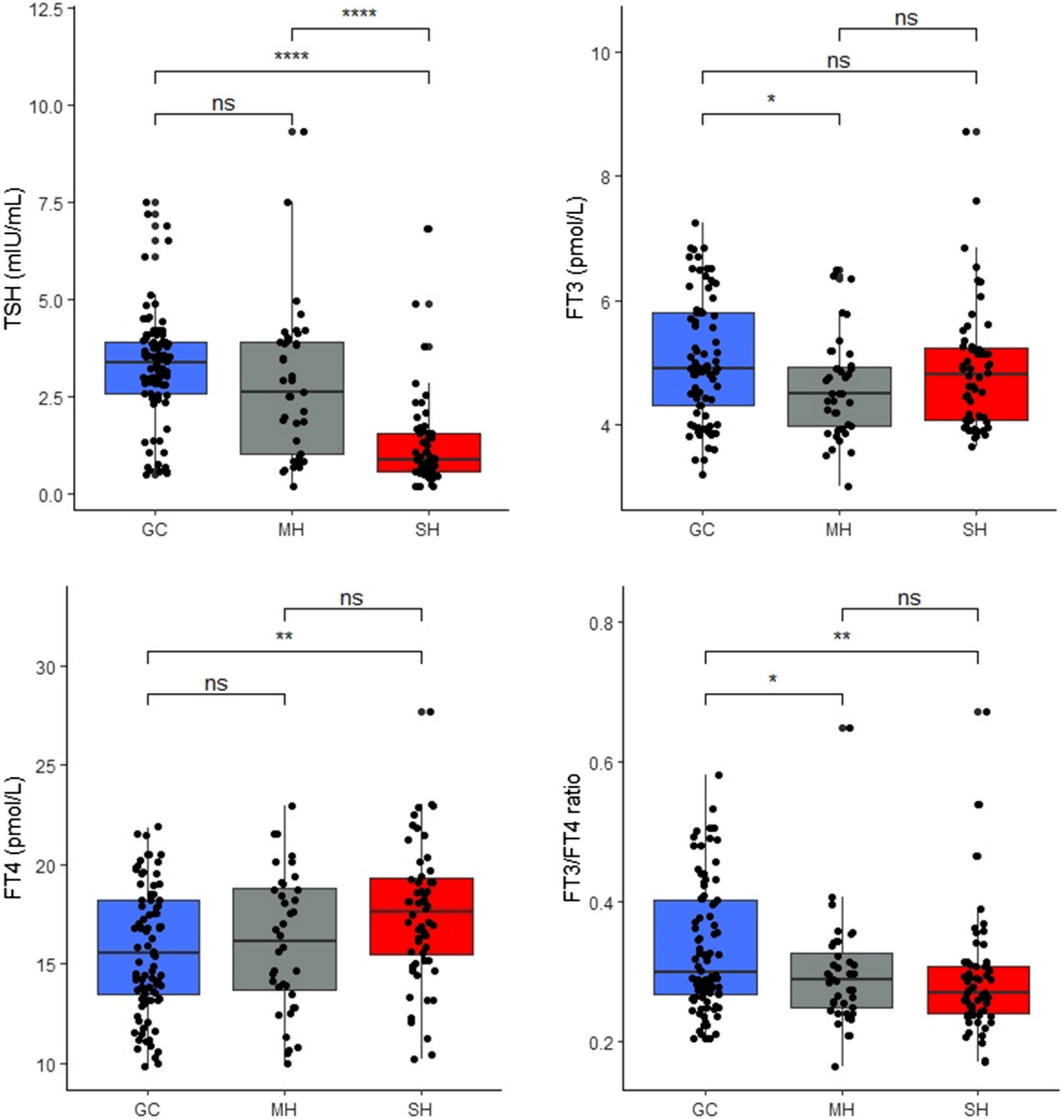
Serum levels of thyroid hormones among T2DM patients. FT3, free triiodothyronine; FT4, free thyroxine; GC, good glycaemic controls; MH, moderate hyperglycaemia; SH, severe hyperglycaemia; TSH, thyroid‐stimulating hormone. *p < .05, **p < .01, ***p < .0001.

The serum level of TSH was significantly lower in T2DM patients with severe hyperglycaemia compared to those with moderate hyperglycaemia and the good glycaemic controls (*p <* .0001). FT3 was significantly lower in MH compared to GC patients (*p <* .05). FT3 did not differ significantly between SH and GC (*p* > .05) or between SH and MH (*p* > .05). The serum level of FT4 was significantly higher SH patients compared to the good glycaemic controls (*p <* .01). FT4 did not differ significantly between GC and MH (*p* > .05) or between MH and SH (*p* > .05). Compared to the GC group, FT3/FT4 ratio was significantly lower in MH (*p* < .05) and SH (*p* < .01) patients (Figure 1).

### 3.4 Association between thyroid hormones and hyperglycaemia

Hyperglycaemia was high (51.0%) among patients with first quartile of TSH (<1.35 mIU/L). Hyperglycaemia however decreased with increasing tertiles of TSH. Similarly, patients with first tertile FT3 had higher prevalence of hyperglycaemia (39.0%). Hyperglycaemia there reduced with increasing tertiles of FT3. It was however noted that hyperglycaemia was uniformly distributed across the tertiles of FT4 and FT3/FT4 ratio.

In a multivariate logistic regression analysis, the first quartiles of TSH [aOR = 10.51, 95% CI (4.04–17.36), *p* < .0001] and FT3 [aOR = 2.77, 95% CI (1.11–6.92), *p* = .0290] were significantly and independently associated with increased odds of hyperglycaemia. Table 3 displays the association between thyroid hormones and hyperglycaemia. Table 3 displays the association between thyroid hormones and hyperglycaemia.

**TABLE 3:** Association between thyroid hormones and hyperglycaemia.

Variables	Total (*n* = 192)	Good glycaemic control (*n* = 92)	Hyperglycaemia (*n* = 100)	aOR	*p*‐Value
*TSH (mIU/L)*					
T1 (<1.35)	62	11 (12.0)	51 (51.0)	10.51 (4.04–17.36)	**<.0001**
T2 (1.35–3.43)	66	36 (39.1)	30 (30.0)	Ref (1)	–
T3 (>3.43)	64	45 (48.9)	19 (19.0)	0.98 (0.36–2.42)	.8850
*FT3 (pmol/L)*					
T1 (<4.41)	63	24 (26.1)	39 (39.0)	2.77 (1.11–6.92)	**.0290**
T2 (4.41–5.13)	67	34 (37.0)	33 (33.0)	Ref (1)	–
T3 (>5.13)	62	34 (37.0)	28 (28.0)	0.71 (0.27–184)	.4820
*FT4 (pmol/L)*					
T1 (<14.50)	63	40 (43.5)	23 (23.0)	0.63 (0.21–1.92)	.4250
T2 (14.50–18.10)	66	28 (30.4)	38 (38.0)	Ref (1)	–
T3 (>18.10)	63	24 (26.1)	39 (39.0)	0.90 (0.35–2.33)	.8310
*FT3/FT4 ratio*					
T1 (<0.36)	63	40 (43.5)	23 (23.0)	1.22 (0.45–3.27)	.6860
T2 (0.26–0.32)	66	28 (30.4)	38 (38.0)	Ref (1)	–
T3 (>0.32)	63	24 (26.1)	39 (39.0)	0.85 (0.27–2.64)	.7860

*Note*: Bold values indicate statistically significant *p*‐value (*p* < .05).

Abbreviations: aOR, adjusted odd ratio, model adjusted for age, gender, ethnicity, education level, type of medication, duration of diabetes and BMI; FT3, free triiodothyronine; FT4, free thyroxine; GC, glycaemic controls; MH, moderate hyperglycaemia; SH, severe hyperglycaemia; T1, first tertile; T2, second tertile; T3, third tertile; TSH, thyroid‐stimulating hormone.

## 4 DISCUSSION

Thyroid dysfunction is the most common endocrinopathy that coexist with diabetics and it has and may influence dysregulation of glycaemic control.^10^, ^26^, ^27^ The development of diabetes complications is known to be dependent on the magnitude, duration and also frequency of hyperglycaemic events,^28^, ^29^ which may be influenced by thyroid dysfunction. In this study, we observed that thyroid dysfunction increases with hyperglycaemia among T2DM patients. The first tertiles of TSH and T3 were independently associated with increased odds of hyperglycaemia.

This study observed decreased TSH levels, increased FT4 and almost unchanged FT3 levels in MH and SH patients compared to the good glycaemic controls. Different levels of TSH have been ascribed to modification of the production and secretion of thyrotrophin releasing hormone by the hypothalamus.^10^, ^30^ TRH and TSH play a key role in the secretion and maintenance of circulating thyroid hormones which control glucose homeostasis and eventually glycaemic levels. There is also what is called thyroid hormone‐binding inhibitor (THBI) which according to Suzuki et al.^31^ contributed to the abnormal thyroid hormone levels in diabetes. THBI is an inhibitor of the extra thyroidal conversion enzyme (5′‐deiodinase) of T4 to T3. The presence of THBI impedes the peripheral conversion of T4 to T3 and thus affecting the levels of T4. This probably accounted for the elevated levels of FT4 and normal or low normal levels of FT3 of those with hyperglycaemia (SH and MH) compared to the good glycaemic controls in this study. The imbalanced hormonal levels as a consequence of diabetes and increased stress levels largely affect the mechanisms that regulates plasma glucose. Intestinal absorption of glucose, changes in circulating insulin levels, increased insulin resistance, hepatic production and peripheral tissues uptake of glucose have been reported as regulatory mechanisms that are affected by thyroid hormones.^10^, ^27^, ^32^

In this current study, overall thyroid dysfunction among T2DM patients was 7.8%, lower than 16.2% and 19.6% reported in Belagavi and South India, respectively.^33^, ^34^ Similar studies from North India, Egypt and Greek also reported 28%, 29% and 12.3% prevalence of thyroid dysfunction among T2DM patients, respectively.^35^, ^36^, ^37^ Moreover, 19.2% of those with hyperglycaemia (SH and MH) had thyroid dysfunction compared to 5.4% of the good glycaemic controls. This observation concurs with previous studies by Jali et al. and Elgazar et al. which also reported increasing trend of thyroid dysfunction with poor glycaemic control.^33^, ^35^ There were 6.7% subclinical hypothyroidism among patients with hyperglycaemia (MH and SH) and 5.4% for the good glycaemic controls. Subclinical hyperthyroidism recorded 10.8% for patients with hyperglycaemia while none was found in the good glycaemic controls. There was 1.7% of primary hyperthyroidism in patients with hyperglycaemia (MH and SH) and none among the good glycaemic controls. A similar study in Ghana by Tagoe et al.^26^ estimated 10.1% thyroid dysfunction in patients with hyperglycaemia and 5.1% among the good glycaemic controls. In a related study by Rai et al. and Udiong et al.^5^, ^38^ prevalence of thyroid dysfunction was found to be 46.5% among Nigerian diabetic patients. In other studies, elsewhere in the world, prevalence of 10.8% and 26.7% thyroid dysfunction were estimated^10^, ^27^ respectively among diabetic patients. The discrepancies in the prevalence rate of thyroid dysfunction observed in diabetics is known to be influenced in part by the various medications administered to the patients, varying levels of sustained hyperglycaemia, frequency and duration of hyperglycaemia which eventually affect the hypothalamus–anterior–pituitary–thyroid axis and glycation of some proteins.^10^, ^32^ Some oral hypoglycaemic agents (OHA) such as phenylthiourea have been discovered to increase TSH and suppress FT4 and T4 levels.^32^, ^39^, ^40^, ^41^

Hyperglycaemia is known to be a risk factor for the development of microvascular complications such as retinopathy and disturbances in the endocrine system which affect the thyroid hormones in diabetes.^41^, ^42^ The hyperglycaemic patients in this study had significantly higher fasting blood glucose and HbA1c compared to the good glycaemic controls. More so, Stratton et al.^43^ stated that reduction in HbA1c in any amount is more likely to lower the risk of diabetic complications and that the lowest risk is found in patients with HbA1c values in the normal range < 6.0%.^41^, ^44^ Hence, tight good glycaemic control is invaluable in arresting the development of thyroid disorders and other complications and probably achieving some significant level of regression in the disease.^2^, ^6^

Anthropometric indices such as BMI were fairly high among all the study groups. However, when the BMI of those with hyperglycaemia were compared to that of the good glycaemic controls, they were significantly higher than the BMI of the good glycaemic controls with elevated systolic pressure (*p* = .040). According to the work done by Azevedo and Alla et al.^45^ increasing obesity has a deteriorating effect on glycaemic control and hence high HbA1c results due to fast glycosylation of haemoglobin and other proteins. This work did not record statistically significant difference in TSH with varying BMI although BMI has a link with glycaemic control which correlates with thyroid function.

This study had few limitations. First, the study was conducted with relatively low sample size. Second, although none of the participants were on thyroid medications, we could not obtain history and pattern of thyroid therapy. The findings from this study however confirms previous findings.

## 5 CONCLUSION

There is a high prevalence of thyroid dysfunction among Type 2 diabetics and the prevalence increases with hyperglycaemia. Reduced TSH and T3 may worsen glycaemic control. Periodic monitoring of thyroid function should be incorporated into management guidelines among T2DM patients in Ghana.

Future investigations should incorporate a relatively larger sample size to effectively evaluate the effects of thyroid dysfunction on glycaemic control. The history and pattern of thyroid therapy should be obtained to determine its association with glycaemic control among Type 2 diabetic patients.

## AUTHOR CONTRIBUTIONS

**Samuel Asamoah Sakyi:** Conceptualization (equal); data curation (equal); formal analysis (equal); investigation (equal); methodology (equal); project administration (equal); supervision (equal); validation (equal); writing – original draft (equal); writing – review and editing (equal). **Bright Ameyaw:** Conceptualization (equal); data curation (equal); formal analysis (equal); investigation (equal); methodology (equal); project administration (equal); validation (equal); visualization (equal); writing – original draft (equal); writing – review and editing (equal). **Edwin Ferguson Laing:** Conceptualization (equal); data curation (equal); methodology (equal); supervision (equal); visualization (equal); writing – review and editing (equal). **Richard Anthony:** Conceptualization (equal); data curation (equal); project administration (equal); validation (equal); visualization (equal). **Richard K. Dadzie Ephraim:** Data curation (equal); formal analysis (equal); investigation (equal); methodology (equal); validation (equal); visualization (equal); writing – review and editing (equal). **Alfred Effah:** Data curation (equal); formal analysis (equal); writing – original draft (equal); writing – review and editing (equal). **Afia Agyapomaa Kwayie:** Data curation (equal); formal analysis (equal); visualization (equal); writing – original draft (equal); writing – review and editing (equal). **Ebenezer Senu:** Data curation (equal); formal analysis (equal); methodology (equal); validation (equal); visualization (equal); writing – original draft (equal); writing – review and editing (equal). **Enoch Odame Anto:** Data curation (equal); formal analysis (equal); investigation (equal); methodology (equal); validation (equal); visualization (equal); writing – original draft (equal); writing – review and editing (equal). **Emmanuel Acheampong:** Data curation (equal); formal analysis (equal); validation (equal); visualization (equal); writing – review and editing (equal). **Bright Oppong Afranie:** Data curation (equal); formal analysis (equal); visualization (equal); writing – original draft (equal); writing – review and editing (equal). **Benjamin Amoani:** Conceptualization (equal); data curation (equal); formal analysis (equal); supervision (equal); writing – original draft (equal); writing – review and editing (equal). **Stephen Opoku:** Data curation (equal); formal analysis (equal); methodology (equal); validation (equal); visualization (equal); writing – original draft (equal); writing – review and editing (equal).

## FUNDING INFORMATION

This research did not receive any specific grant from funding agencies in the public, commercial or not‐for‐profit sectors.

## CONFLICT OF INTEREST STATEMENT

The authors declare that they have no known competing financial interests or personal relationships that could have appeared to influence the work reported in this paper.

## Data Availability

All data generated or analysed during this study are included in this published article and its supplementary information files data and can be requested from corresponding author.

## References

[1] WHO . Global report on diabetes. 2016. https://www.who.int/publications/i/item/9789241565257

[2] Association, A. D . Standards of medical Care in Diabetes—2014. Diabetes Care. 2013;37(Supplement_1):S14‐S80. 10.2337/dc14-S014 24357209

[3] Nauck MA . A critical analysis of the clinical use of incretin‐based therapies: the benefits by far outweigh the potential risks. Diabetes Care. 2013;36(7):2126‐2132. 23645884 10.2337/dc12-2504 PMC3687264

[4] Danquah I , Bedu‐Addo G , Terpe K‐J , et al. Diabetes mellitus type 2 in urban Ghana: characteristics and associated factors. BMC Public Health. 2012;12(1):210. 22429713 10.1186/1471-2458-12-210 PMC3364878

[5] Udiong CEJ , Udoh AE , Etukudoh ME . Evaluation of thyroid function in diabetes mellitus in Calabar, Nigeria. Indian J Clin Biochem. 2007;22(2):74‐78. 23105687 10.1007/BF02913318 PMC3453810

[6] Chen G , Wu J , Lin Y , et al. Associations between cardiovascular risk, insulin resistance, β‐cell function and thyroid dysfunction: a cross‐sectional study in she ethnic minority group of Fujian Province in China. Eur J Endocrinol. 2010;163(5):775‐782. 20798227 10.1530/EJE-10-0710

[7] Jörns A , Tiedge M , Lenzen S . Thyroxine induces pancreatic beta‐cell apoptosis in rats. Diabetologia. 2002;45(6):851‐855. 12107729 10.1007/s00125-002-0842-5

[8] Kalra S , Aggarwal S , Khandelwal D . Thyroid dysfunction and type 2 diabetes mellitus: screening strategies and implications for management. Diabetes Ther. 2019;10(6):2035‐2044. 31583645 10.1007/s13300-019-00700-4 PMC6848627

[9] Hage M , Zantout MS , Azar ST . Thyroid disorders and diabetes mellitus. J Thyroid Res. 2011;2011:439463. 21785689 10.4061/2011/439463 PMC3139205

[10] Anveetha P , Rao KP , Chittimoju VK . Research Article Biological Sciences. www.ijpbsonline.com

[11] Uppal V , Vij C , Bedi GK , Vij A , Banerjee BD . Thyroid disorders in patients of type 2 diabetes mellitus. Indian J Clin Biochem. 2013;28(4):336‐341. 24426234 10.1007/s12291-012-0293-9 PMC3783922

[12] Ximenes HM , Lortz S , Jörns A , Lenzen S . Triiodothyronine (T3)‐mediated toxicity and induction of apoptosis in insulin‐producing INS‐1 cells. Life Sci. 2007;80(22):2045‐2050. 17408701 10.1016/j.lfs.2007.03.001

[13] Ximenes HMA , Hirata AE , Rocha MS , Curi R , Carpinelli AR . Propionate inhibits glucose‐induced insulin secretion in isolated rat pancreatic islets. Cell Biochem Funct. 2007;25(2):173‐178. 16444779 10.1002/cbf.1297

[14] Mathers CD , Loncar D . Projections of global mortality and burden of disease from 2002 to 2030. PLoS Med. 2006;3(11):e442. 17132052 10.1371/journal.pmed.0030442 PMC1664601

[15] Association, A. D . 2. Classification and diagnosis of diabetes. Diabetes Care. 2014;38(Supplement_1):S8‐S16. 10.2337/dc15-S005 25537714

[16] Thomas J , Monaghan T . Oxford Handbook of Clinical Examination and Practical Skills. Oxford University Press; 2014.

[17] Owiredu E‐W , Dontoh E , Essuman SES , Bazanfara BB . Demographic and lifestyle predictors of prehypertension: a cross‐sectional study among apparently healthy adults in Kumasi, Ghana. BioMed Res Int. 2019;2019:1764079. 31179316 10.1155/2019/1764079 PMC6507075

[18] Baumgartner C , Blum MR , Rodondi N . Subclinical hypothyroidism: summary of evidence in 2014. Swiss Med Wkly. 2014;144(5152):w14058. 25536449 10.4414/smw.2014.14058

[19] Hegedüs L , Bianco AC , Jonklaas J , Pearce SH , Weetman AP , Perros P . Primary hypothyroidism and quality of life. Nat Rev Endocrinol. 2022;18(4):230‐242. 35042968 10.1038/s41574-021-00625-8 PMC8930682

[20] Peeters RP . Subclinical hypothyroidism. N Engl J Med. 2017;376(26):2556‐2565. 28657873 10.1056/NEJMcp1611144

[21] Nygaard B . Hyperthyroidism (primary). BMJ Clin Evid. 2010;2010:0611. PMC3275323 21418670

[22] Siu C‐W , Yeung C‐Y , Lau C‐P , Kung AWC , Tse H‐F . Incidence, clinical characteristics and outcome of congestive heart failure as the initial presentation in patients with primary hyperthyroidism. Heart. 2007;93(4):483‐487. 17005710 10.1136/hrt.2006.100628 PMC1861478

[23] Mellanby RJ , Jeffery ND , Gopal MS , Herrtage ME . Secondary hypothyroidism following head trauma in a cat. J Feline Med Surg. 2005;7(2):135‐139. 15771951 10.1016/j.jfms.2004.08.002 PMC10822260

[24] Studer H , Wyss F , Jff HW . A TSH reserve test for detection of mild secondary hypothyroidism. J Clin Endocrinol Metabol. 1964;24(10):965‐975. 10.1210/jcem-24-10-965 14228535

[25] Cohen Y , Cohen J . Statistics and Data with R: An Applied Approach Through Examples. John Wiley & Sons; 2008.

[26] Aryee NA , Tagoe EA . Association of thyroid‐stimulating hormone and thyroid hormones with lipid profile in Ghanaian Euthyroid patients with type 2 diabetes. 2014. https://citeseerx.ist.psu.edu/document?repid=rep1&type=pdf&doi=8c4d07d15c2d191fb2244c06dd583dd60b082c22

[27] Johnson JL . Diabetes control in thyroid disease. Diabetes Spectrum. 2006;19(3):148‐153.

[28] Manaviat MR , Rashidi M , Afkhami‐Ardekani M , Shoja MR . Prevalence of dry eye syndrome and diabetic retinopathy in type 2 diabetic patients. BMC Ophthalmol. 2008;8(1):10. 18513455 10.1186/1471-2415-8-10 PMC2435518

[29] Shaw JE , Sicree RA , Zimmet PZ . Global estimates of the prevalence of diabetes for 2010 and 2030. Diabetes Res Clin Pract. 2010;87(1):4‐14. 19896746 10.1016/j.diabres.2009.10.007

[30] Perros P , McCrimmon RJ , Shaw G , Frier BM . Frequency of thyroid dysfunction in diabetic patients: value of annual screening. Diabet Med. 1995;12(7):622‐627. 7554786 10.1111/j.1464-5491.1995.tb00553.x

[31] Suzuki Y , Nanno M , Gemma R , Tanaka I , Taminato T , Yoshimi T . The mechanism of thyroid hormone abnormalities in patients with diabetes mellitus. Nihon Naibunpi Gakkai Zasshi. 1994;70(4):465‐470. 7958096 10.1507/endocrine1927.70.4_465

[32] Vij V , Chitnis P , Gupta VK . Evaluation of thyroid dysfunction among type II diabetic patients. Ijpbs. 2012;2(4):150‐155.

[33] Jali MV , Kambar S , Jali SM , Pawar N , Nalawade P . Prevalence of thyroid dysfunction among type 2 diabetes mellitus patients. Diabetes Metab Syndr Clin Res Rev. 2017;11:S105‐S108. 10.1016/j.dsx.2016.12.017 28057505

[34] Reddy N , Pradeep TVS , Tirupati S , Sarathi V , Kumar D . Thyroid dysfunction and its association with microvascular complications in patients with type 2 diabetes mellitus in South India. Diabetes Metab Syndr Clin Res Rev. 2020;14(4):615‐617. 10.1016/j.dsx.2020.05.005 32422445

[35] Elgazar EH , Esheba NE , Shalaby SA , Mohamed WF . Thyroid dysfunction prevalence and relation to glycemic control in patients with type 2 diabetes mellitus. Diabetes Metab Syndr Clin Res Rev. 2019;13(4):2513‐2517. 10.1016/j.dsx.2019.07.020 31405670

[36] Ozair M , Noor S , Raghav A , Siddiqi SS , Chugtai AM , Ahmad J . Prevalence of thyroid disorders in north Indian type 2 diabetic subjects: a cross sectional study. Diabetes Metab Syndr Clin Res Rev. 2018;12(3):301‐304. 10.1016/j.dsx.2017.12.016 29279270

[37] Papazafiropoulou A , Sotiropoulos A , Kokolaki A , Kardara M , Stamataki P , Pappas S . Prevalence of thyroid dysfunction among greek type 2 diabetic patients attending an outpatient clinic. J Clin Med Res. 2010;2(2):75‐78. 21811523 10.4021/jocmr2010.03.281w PMC3140882

[38] Rai S , Kumar JA , Prajna K , et al. Thyroid function in type 2 diabetes mellitus and in diabetic nephropathy. J Clin Diagn Res. 2013;7(8):1583‐1585. 24086845 10.7860/JCDR/2013/6216.3299 PMC3782902

[39] Logmans SC , Jöbsis AC . Thyroid‐associated antigens in routinely embedded carcinomas. Possibilities and limitations studied in 116 cases. Cancer. 1984;54(2):274‐279. 6202390 10.1002/1097-0142(19840715)54:2<274::aid-cncr2820540215>3.0.co;2-1

[40] Wild S , Roglic G , Green A , Sicree R , King H . Global prevalence of diabetes: estimates for the year 2000 and projections for 2030. Diabetes Care. 2004;27(5):1047‐1053. 15111519 10.2337/diacare.27.5.1047

[41] WHO . Definition and Diagnosis of Diabetes Mellitus and Intermediate Hyperglycemia: Report of a WHO/IDF Consultation. World Hearth Organisation; 2006.

[42] Tumosa N . Eye disease and the older diabetic. Clin Geriatr Med. 2008;24(3):515‐527. 18672186 10.1016/j.cger.2008.03.002

[43] Stratton IM , Adler AI , Neil HAW , et al. Association of glycaemia with macrovascular and microvascular complications of type 2 diabetes (UKPDS 35): prospective observational study. BMJ. 2000;321(7258):405‐412. 10938048 10.1136/bmj.321.7258.405 PMC27454

[44] Whiting DR , Guariguata L , Weil C , Shaw J . IDF diabetes atlas: global estimates of the prevalence of diabetes for 2011 and 2030. Diabetes Res Clin Pract. 2011;94(3):311‐321. 22079683 10.1016/j.diabres.2011.10.029

[45] Azevedo M , Alla S . Diabetes in sub‐saharan Africa: Kenya, Mali, Mozambique, Nigeria, South Africa and Zambia. Int J Diabetes Dev Ctries. 2008;28(4):101‐108. 20165596 10.4103/0973-3930.45268 PMC2822152

